# Draft Genome Sequence of *Acetobacter aceti* Strain 1023, a Vinegar Factory Isolate

**DOI:** 10.1128/genomeA.00550-14

**Published:** 2014-06-05

**Authors:** John E. Hung, Christopher P. Mill, Sandra W. Clifton, Vincent Magrini, Ketaki Bhide, Julie A. Francois, Aaron E. Ransome, Lucinda Fulton, Jyothi Thimmapuram, Richard K. Wilson, T. Joseph Kappock

**Affiliations:** a Department of Chemistry, Washington University in St. Louis, St. Louis, Missouri, USA; b The Genome Institute, Washington University School of Medicine, St. Louis, Missouri, USA; c Department of Genetics, Washington University School of Medicine, St. Louis, Missouri, USA; d Bioinformatics Core, Discovery Park, Purdue University, West Lafayette, Indiana, USA; e Department of Biochemistry, Purdue University, West Lafayette, Indiana, USA

## Abstract

The genome sequence of *Acetobacter aceti* 1023, an acetic acid bacterium adapted to traditional vinegar fermentation, comprises 3.0 Mb (chromosome plus plasmids). *A. aceti* 1023 is closely related to the cocoa fermenter *Acetobacter pasteurianus* 386B but possesses many additional insertion sequence elements.

## GENOME ANNOUNCEMENT

Acetic acid bacteria (AAB) are acidophilic aerobic alphaproteobacteria with many uses in food processing (1, 2). *Acetobacter aceti* strain 1023, a traditional rice vinegar mash surface isolate (3), was used in pioneering studies of AAB physiology (4). The continual selection of vinegar strains has favored acetic acid/ethanol resistance traits and disfavored wasteful overoxidation, in which acetic acid is lost as CO_2_ (5). Whole-genome sequencing of *A. aceti* 1023 was used to identify adaptations in this highly domesticated vinegar strain.

*A. aceti* 1023 was propagated at 30°C in yeast-peptone-dextrose medium supplemented with 2% ethanol. Genomic DNA was used to prepare plasmid (4.1- and 6.1-kb inserts in plasmid pOTW13) and fosmid (40-kb inserts in pCC-FOS1) libraries, as previously described (6, 7). Using PCAP (8), paired-end Sanger reads were assembled (28,731 reads, 76% input) into 337 contigs >1 kb (total, 3.2 Mb; *N*_50_, 17,669 bp), as was disclosed in a preliminary form (9).

Genomic DNA libraries were analyzed by 454 GS-FLX pyrosequencing using both fragment (564,984 reads, 140 Mb total) and mate-pair (3-kb insert; 468,069 reads, 66 Mb total) libraries. A hybrid assembly of Sanger and 454 reads using Newbler (version 2.9) furnished 33 scaffolds composed of 193 contigs (>0.5 kb) and 3.0 Mb total sequence at 72-fold coverage. The scaffolds were ordered with Mauve (version 2.3.1) (10), using the complete genome sequence of *Acetobacter pasteurianus* 386B (11) as the template. The NCBI Prokaryotic Genome Annotation Pipeline (version 2.5) and BLASTn analysis predicted 2,650 open reading frames, 66 pseudogenes, and 47 functional RNAs. At least eight scaffolds (0.07 Mb total) appeared to originate from plasmids, as judged by the presence of *repBA* and plasmid partitioning genes. As is typical for the low-copy-number AAB “cryptic” plasmids (12), the plasmid scaffolds contain few genes that clearly confer a phenotype.

A phylogenetic analysis of AAB GroEL sequences (13) grouped *A. aceti* 1023 with *A. pasteurianus* and *Acetobacter pomorum*, not *A. aceti* NBRC 14818 or ATCC 23746. Central carbon metabolism is more straightforward in *A. aceti* 1023 and *A. pasteurianus* strains, which use a specialized citric acid cycle containing *aarC* (14), than in *A. aceti* NBRC 14818, which has greater metabolic versatility (15–17). As judged by gene synteny and sequence similarity, *A. aceti* 1023 has a particularly close relationship to *A. pasteurianus* 386B, a cocoa fermenter (11). However, *A. pasteurianus* 386B lacks numerous insertion sequence (IS) elements present in the vinegar strains *A. aceti* 1023 and *A. pasteurianus* NBRC 3283 (18). As anticipated from Southern blots (19–21), *A. aceti* 1023 contains IS*1380*, IS*1452*, and IS*12538*, with minimal copy numbers of 64, 4, and 1, respectively. The adaptation of a common ancestor to different fermentation milieux involved divergent histories of transposable element acquisition in *A. aceti* 1023 and *A. pasteurianus* 386B.

### Nucleotide sequence accession numbers.

This whole-genome shotgun project has been deposited in DDBJ/ENA/GenBank under the accession no. JEOA00000000. The version described in this paper is the first version, JEOA01000000.
